# Experimental evidence for asymmetric mate preference and aggression: behavioral interactions in a woodrat (*Neotoma*) hybrid zone

**DOI:** 10.1186/1471-2148-13-220

**Published:** 2013-10-04

**Authors:** Quinn R Shurtliff, Peter J Murphy, Jaclyn D Yeiter, Marjorie D Matocq

**Affiliations:** 1 Wildlife Conservation Society, North America Program, Idaho Falls, ID 83402, USA; 2 Department of Natural Resources and Environmental Science, Mailstop 0186, University of Nevada Reno, Reno, NV 89557, USA; 3 Program in Ecology, Evolution, and Conservation Biology, University of Nevada, Reno, NV 89557, USA; 4 Department of Biological Sciences, Idaho State University, Pocatello, ID 83209, USA

**Keywords:** Hybridization, Reproductive isolation, Reproductive character displacement, Reinforcement

## Abstract

**Background:**

Female mate preferences may be under strong selection in zones of contact between closely related species because of greater variation in available mates and the potential costs of hybridization. We studied female mate preferences experimentally in a zone of secondary contact between Desert and Bryant’s Woodrat (*Neotoma lepida* and *N. bryanti*) in the southern foothills of the Sierra Nevada of California. We tested female preference for conspecific versus heterospecific males in paired choice trials in which females could interact freely with males, but males could not interact directly with each other. We compared preferences of females from both allopatric and sympatric sites.

**Results:**

We did not find evidence of the process of reinforcement as assortative preferences were not stronger in sympatry than in allopatry. Mate preferences, however, were asymmetric, with *N. lepida* females mating preferentially with conspecifics and *N. bryanti* females showing no preference by species. Sympatric females were less likely to mate than allopatric females, due in part to an increase in aggressive interactions. However, even in the absence of aggression, courtship led to mating less often in sympatric females, suggesting they were choosier or had lower sexual motivation than allopatric females.

**Conclusions:**

Patterns of mate choice in this woodrat system appear to be strongly impacted by body size and aggressive behavior. In particular, females of the smaller-bodied species rarely interact with the relatively large heterospecific males. In contrast females of the larger-bodied species accept the relatively small heterospecific males. For sympatric animals, rates of aggression were markedly higher than for allopatric animals and reduced affiliative and reproductive behavior in our trials. Sympatric animals are larger and more aggressive, traits that are likely under strong ecological selection across the sharp resource gradient that characterizes the contact zone. However, our results suggest that these traits that are likely favored in competitive interactions between the species also impact reproductive interactions. Combined with our previous findings of post-zygotic isolation in this system, this study suggests that multiple isolating mechanisms contribute to the rate of genetic exchange between these species when they come into contact, and that these mechanisms are the result of selection on traits that are important in a range of ecological and reproductive interactions.

## Background

When closely related species that have diverged in isolation come into secondary contact, we have a unique opportunity to examine the range of pre- and post-zygotic isolating mechanisms that maintain the species boundary, and the degree to which the boundary may still be permeable to gene flow [1,2]. Despite great interest in the process of speciation, we continue to know relatively little concerning the range of isolating mechanisms that characterize particular systems, the order in which isolating mechanisms evolve, and the degree to which they interact under certain environmental conditions [3]. Likewise, the roles of natural and sexual selection in shaping traits that facilitate or constrain gene flow have long been recognized, but the degree to which they act in concert or in sequence is only beginning to be understood [3-7]).

Following secondary contact, it is expected that post-zygotic isolation due to low hybrid fitness will minimize gene flow between species [7]. Lowered fitness in hybrids can be the result of genomic incompatibility between differentiated genomes [8,9], perhaps only made evident under certain environmental conditions [1]. Whether initial genomic differentiation that occurred in allopatry was the result of drift or ecological adaptation [10,11], once genomes are mixed through hybridization, selection has an opportunity to act on novel recombinants. If hybrids have lower fitness, selection should act to minimize behaviors that cause pure parental individuals to incur the costs of hybridization [12,13]. That is, when post-zygotic isolation exists, selection should promote pre-zygotic isolation, perhaps in the form of assortative mating [14,15].

In hybrid zones characterized by low hybrid fitness, traits that determine mate preferences are expected to be under selection [4,16]. However, the same traits that play a role in mating cues, are perhaps also responding to natural selection for their roles in other ecological interactions [4,6,10]. For example, body size is a trait that can be under both natural and sexual selection [17,18]. Large body size is often associated with augmented aggression [19] and the ability to defend territory [20,21]. However, large size accompanied by aggressive behavior may be a deterrent to mating interactions [22]. As such, in a hybrid zone, body size and aggression may respond to aspects of natural selection related to heightened interspecific competition, but these traits may simultaneously play a role in mate choice outcomes.

**Figure 1:**
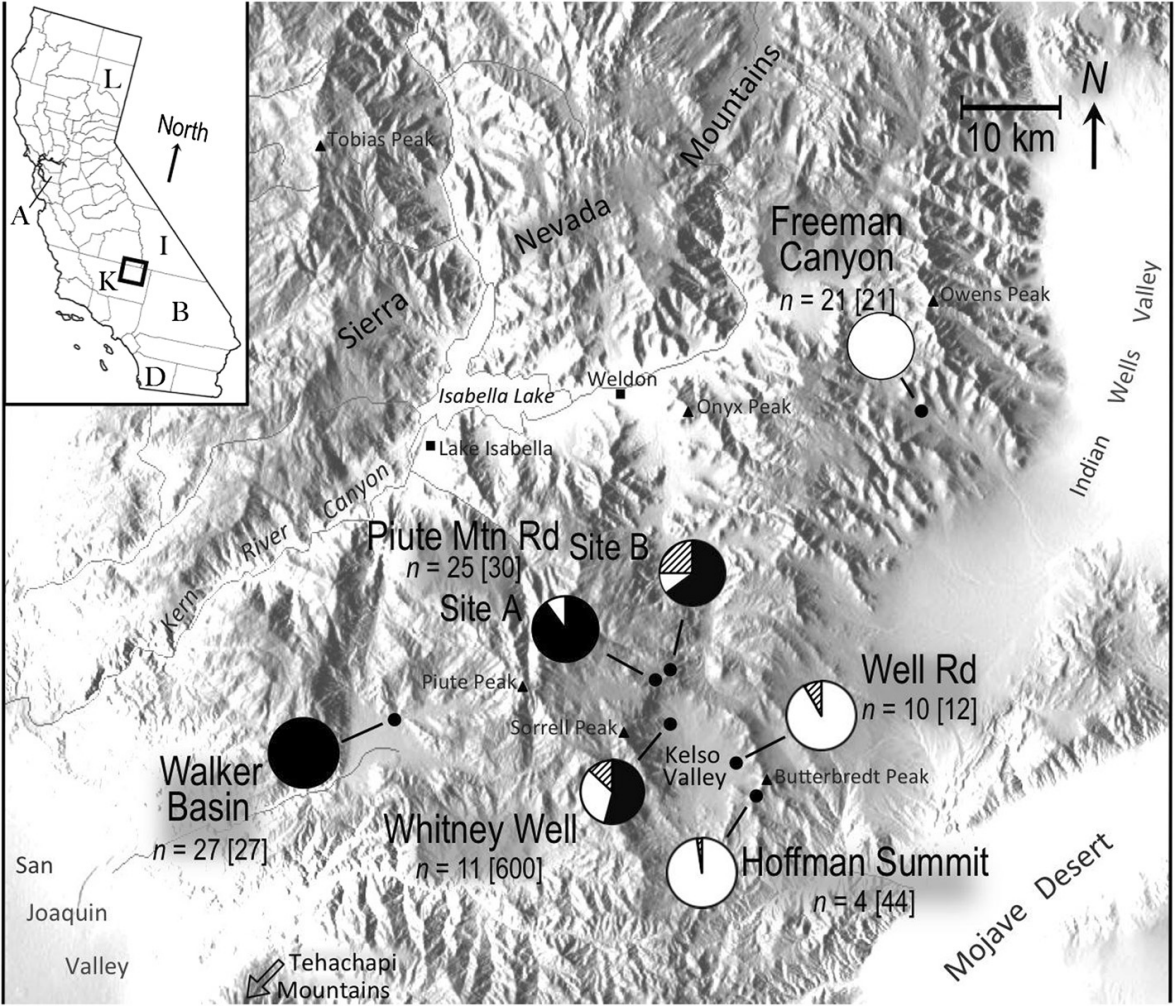
**Location of woodrat contact zone in the Kelso Valley of the southern Sierra Nevada, California where females and males for mate preference trials were trapped from seven localities (●).** Pie charts represent the genotypic proportion of *Neotoma bryanti* (black), *N. lepida* (white), and hybrids (cross-hatch). Sample sizes (*n*) of experimental [and genotyped] individuals are noted with each piechart. Letters in inset indicate counties of origin for specimens that were used as part of an analysis of body mass (A = Alameda, I = Inyo, K = Kern, L = Lassen, B = San Bernadino, and D = San Diego).

We have recently documented strong selection against hybrids (Figure 1, Whitney Well locality) in a narrow hybrid zone between two sister species of woodrats, *Neotoma bryanti* and *N. lepida*. Specifically, in a 4-year demographic study, juvenile hybrids survived the first year at less than half the rate of their purebred counterparts (10% versus 28% [23]). These contrasting patterns of survival at Whitney Well occurred at a sharp environmental transition between relatively mesic coastal/Sierran oak-scrub and the Mojave desert scrub community. In this ecological setting, the species are strongly segregated by habitat type with the large-bodied *N. bryanti* occupying the boulder-strewn, relatively mesic habitat on the west-side of the contact zone and *N. lepida* occupying the desert scrub habitat on the east end of the site [23]. The strong genotype-environment relationship exhibited by the parental classes suggests differential ecological adaptation. As such, it is likely that lowered hybrid fitness is at least partly ecologically-based. Regardless of the source of selection against hybrids, of those that survive, at least some appear to be capable of reproducing because a wide range of recombinant genotypes exist across the zone with approximately 13% of the population being of hybrid origin [23].

Because low hybrid survival could be a source of selection against behaviors that lead to hybridization [12], we sought to understand whether other isolating mechanisms exist in this system, specifically, whether the species exhibit pre-mating isolation in the form of assortative mating. To provide context for our observations of behavioral interactions among individuals in the contact zone, we compare them to observations between individuals from nearby allopatric populations. We expect that contact zone or sympatric populations that have experienced the cost of hybridization (low hybrid survival) are under selection to minimize heterospecific matings. Therefore, purebred individuals should exhibit stronger patterns of assortative mating in sympatry than in allopatry, a pattern consistent with reproductive character displacement due to reinforcement [12,24,25]. Our experimental design focuses on intersexual interactions with females being given a choice to interact with males of either species. For these interactions, males were tethered within their home cages, and thus, visitation by a female offered opportunity for a wide range of behavioral interactions from social-affiliative behaviors, including mating, to social-agonistic and aggressive behaviors.

## Results

### Behavioral interactions: the effects of female taxon in allopatry and sympatry

**Figure 2:**
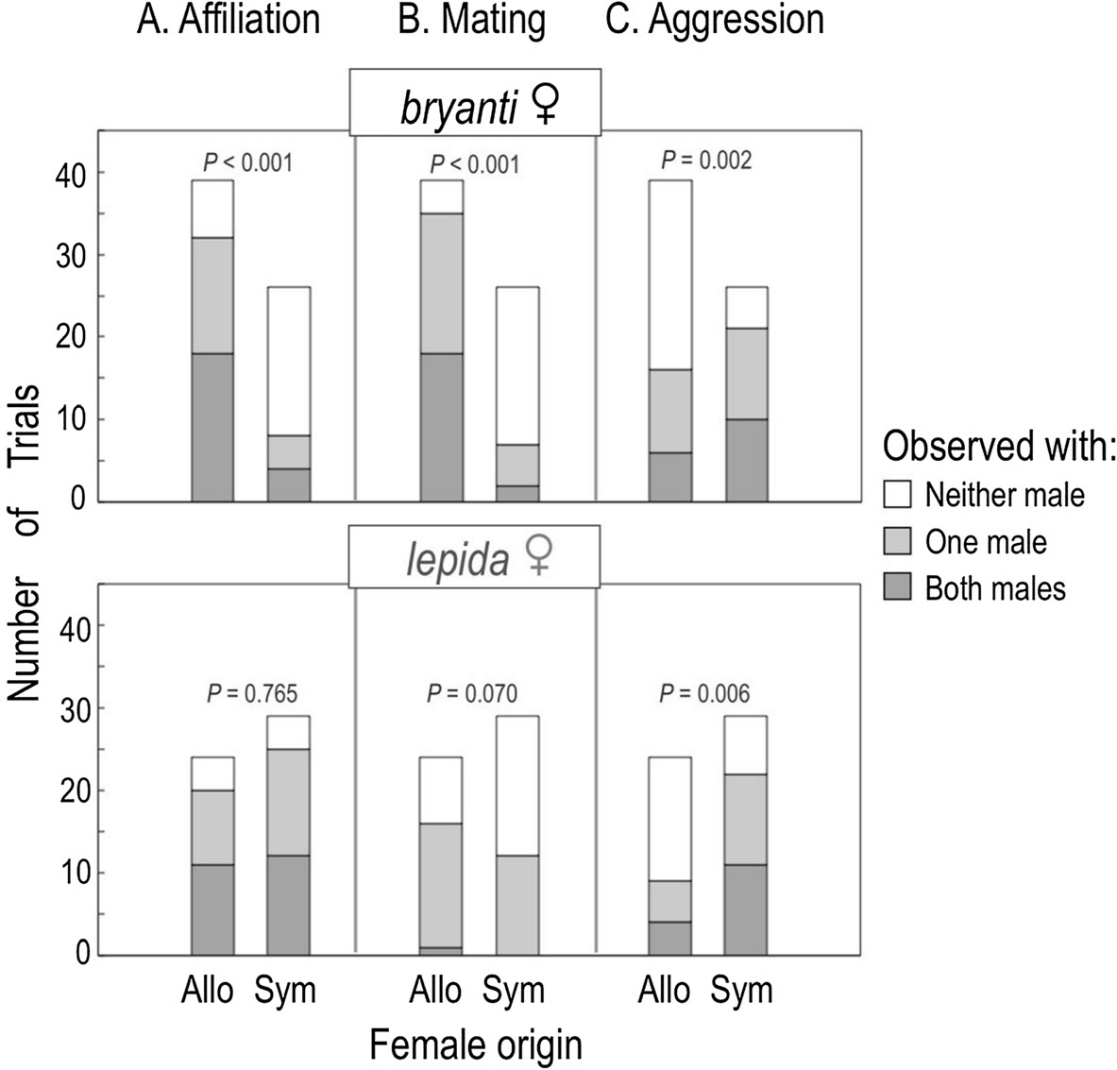
**The number of mate choice trials conducted and activity observed by species and region of origin.** The panels show the occurrence of **(A)** affiliation, **(B)** mating, and **(C)** aggression within trials. The *P*-values are from Cochran-Mantel-Haenszel tests that ask whether the likelihood of the activity occurring with ≥ 1 male was independent of female origin (while controlling for trial number, as each female was used for 2 trials, *seeMethods*).

We conducted 118 trials involving 65 females, with 12 females tested only once. Here we report results from Cochran-Mantel-Haenszel tests $\left(\chi_{\mathrm{cmh}}^{2}\right)$ that test whether the occurrence of the behavior (with at least one male, *i.e*., light and dark regions of bars pooled, Figure 2) is independent of region. The CMH statistic controls for any change in behavior observed from trial 1 to trial 2. Social-affiliation decreased in sympatric versus allopatric trials in *N. bryanti* ($\chi_{\mathrm{cmh}}^{2}=17.1$, *P* < 0.001) but not in *N. lepida* ($\chi_{\mathrm{cmh}}^{2}=0.1$, *P* = 0.765; Figure 2A). Mating activity also declined from allopatry to sympatry in *N. bryanti* ($\chi_{\mathrm{cmh}}^{2}=26.1$, *P* < 0.001) and less sharply in *N. lepida* ($\chi_{\mathrm{cmh}}^{2}=3.3$, *P* = 0.070; Figure 2B). The regional trend in aggressive behavior was opposite that of mating activity. In both species there was an increase in aggression in trials with sympatric females compared to trials with allopatric females (*N.bryanti*: $\chi_{\mathrm{cmh}}^{2}=9.7$, *P* = 0.002; *N.lepida*: $\chi_{\mathrm{cmh}}^{2}=7.7$, *P* = 0.006; Figure 2C). The relationships between region and affiliation, mating activity, or aggression, did not differ from female trial 1 to trial 2 ($\chi_{\mathrm{BD}}^{2}\leq 2.4$, *P* ≥ 0.123). For this reason, we pooled data from all trials in further analysis. When it occurred, regardless of region, mating activity began in the first hour in 96% of the trials. *Neotoma bryanti* females frequently mated with both males during a trial (20 of 65 trials, Figure 2B, darkest bars), more often than did *N. lepida* ($\chi_{\mathrm{cmh}}^{2}=19.0$, *P* < 0.001) and more often in allopatry than in sympatry ($\chi_{\mathrm{cmh}}^{2}=13.0$, *P* < 0.001). Of the *N. bryanti* that mated with both males, half mated first with the conspecific and half first with the heterospecific male.

We conducted additional contingency tests to see whether the link between affiliation and mating, and aggression and the absence of mating, varied by female taxon and region. In encounters with only affiliative behavior (no aggression), mating was less likely in sympatry than in allopatry ($\chi_{\mathrm{cmh}}^{2}=6.7$, *P* = 0.010), and the pattern did not differ by species ($\chi_{\mathrm{BD}}^{2}=0.3$, *P* = 0.556). Trials with only aggression (no affiliation) were more likely in sympatry than in allopatry (14 vs. 4, $\chi_{\mathrm{cmh}}^{2}=2.9$, *P* = 0.088), regardless of female taxon ($\chi_{\mathrm{BD}}^{2}=0.2$, *P* = 0.687). Sympatric females were less likely to mate with a male with whom they had been aggressive than were allopatric females. This was due to *N. bryanti* females whose decline in mating given aggression was larger than for *N. lepida* ($\chi_{\mathrm{BD}}^{2}=3.4$, *P* = 0.065). In *N. bryanti*, aggressive encounters with conspecifics ($\chi_{1}^{2}=15.8$, *P* < 0.001) rather than heterospecifics ($\chi_{1}^{2}=1.1$, *P* = 0.285) produced most of this decline. In *N. lepida*, we never observed mating with heterospecific males when aggression occurred with them; with conspecific males, mating given aggression was rare and did not change in frequency from allopatry to sympatry ($\chi_{1}^{2}=0.1$, *P* = 0.871).

Females collected during the first sampling trip were less likely to mate (23% of trials) than those collected in the second or third trips (81% and 59% of trials, respectively; logistic regression: $\chi_{2}^{2}=9.7$, *P* = 0.008). There was no effect of the season a trial was conducted on the likelihood of a mating ($\chi_{3}^{2}=4.3$, *P* = 0.235) when sampling trip was also included in the model. Because poor condition due to drought may have accounted in part for the low rate of mating initially observed in females trapped in the first sampling trip, we excluded these woodrats and again compared mating activity in sympatric and allopatric females. We still found that sympatric females were less likely to mate in trials ($\chi_{1}^{2}=4.3$, *P* = 0.038).

### Intensity of female activity with conspecific and heterospecific males

**Figure 3:**
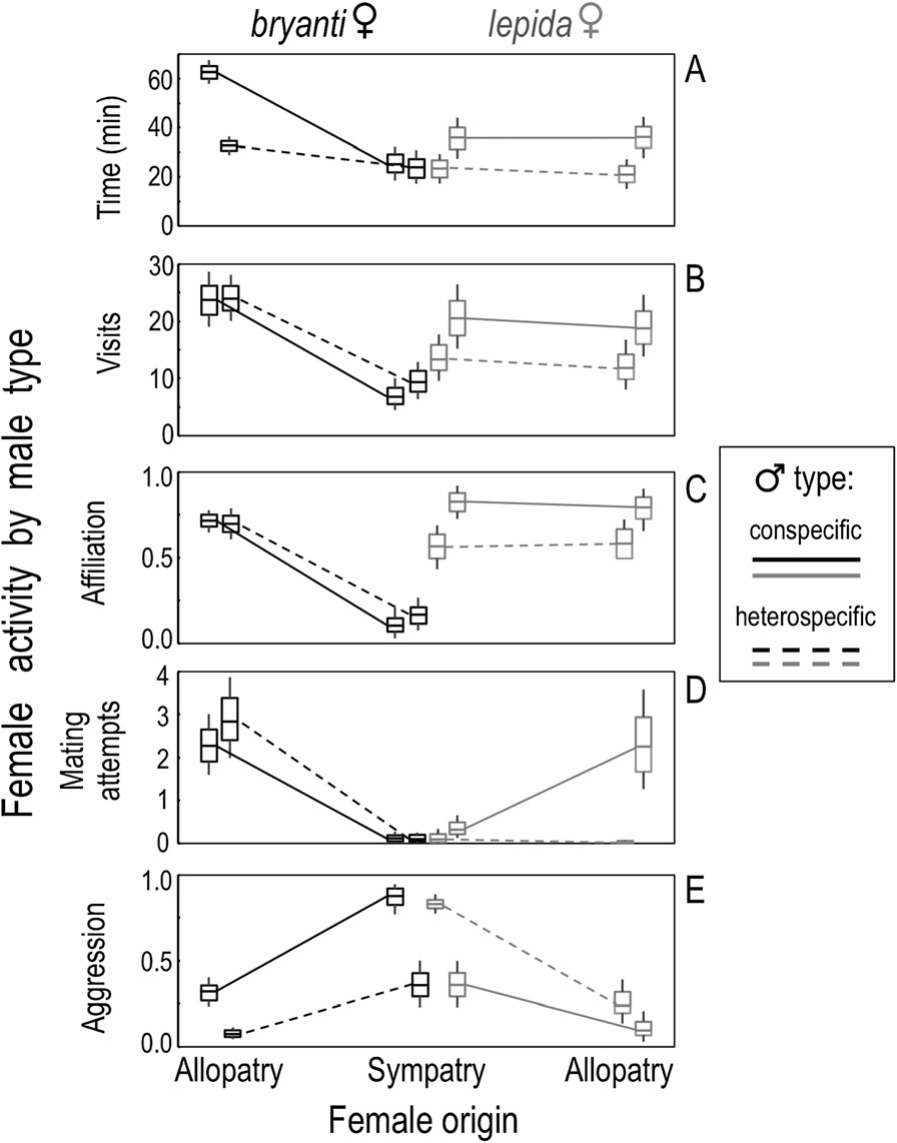
**Female behavior by species and region with conspecific and heterospecific males.** The 5 responses include: **(A)** time with a male (entry tube plus cage, Figure 6), **(B)** visits to a male’s cage, **(C)** affiliation, **(D)** mating attempts, and **(E)** aggression. Responses **(C)** and **(E)** were the proportion of a female’s 2 trials where affiliation or aggression was observed. Each box plot shows the distribution of least squares means (median and inter-quartile range) from 1000 anova models (whiskers represent 10% and 90% values). Each model analyzed a female’s response with either conspecific or heterospecific males on a random subsample of all trials to ensure that focal observations were independent (see *Methods: Data analysis*). The models for cage visits, affiliation, mating attempts, and aggression were run on transformed values, but back-transformed values are shown.

With respect to activity prior to mating, *N. bryanti* females declined from allopatry to sympatry in time, visits, and affiliation with conspecific males, whereas *N. lepida* was regionally constant in these responses (Figure 3A-C, solid lines), differences suggestive but not significant in our analyses (Table 1A, species-by-region *P* ≤ 0.05 in 50-63% of models). With heterospecific males, a similar but weaker difference was evident between females by species in time, visits, and affiliation: a regional decline in *N. bryanti* and no change in *N. lepida* (species-by-region, *P* ≤ 0.05 in 12-39% of models). Overall, *N. lepida* showed consistently more affiliative behavior with conspecific males than *N. bryanti* (Figure 3C, median boxplot height for conspecific males; Table 1A, *P* ≤ 0.05 in 87% of models). Females did not differ in their behavior from trial 1 to 2, and male relative mass did not consistently affect any female response variable (Table 1A,B). Mating activity with conspecific males by females of both species declined strongly from allopatry to sympatry (Figure 3D, solid lines; Table 1A, 99% region effect). Mating activity with heterospecifics declined from sympatry to allopatry in *N. bryanti*, but was low in both regions in *N. lepida* (Figure 3D, dashed lines; Table 1B, *P* ≤ 0.05 for species in 90% and species-by-region in 96% of models).

**Table 1:** The strength of female interactions in mate choice trials by species and region with (A) conspecific males and (B) heterospecific males

	**Female interaction by male class (trials per bootstrap replicate)**					
**Fixed effect**	**df**	**Time (58–59)**	**Visits (57–59)**	**Affiliation (40–53)**	**Mating attempts (59)**	**Aggression (40–52)**
**A. With conspecific males**						
Female species*	1	5	12	*87*	2	*75*
Female region*	1	67	32	45	***99***	*86*
Species x region*	1	63	50	61	3	3^b^
Male relative mass^a^	1	1	1	24	13	1
Trial number	1	1	1	.	1	.
**B. With heterospecific males**						
Female species*	1	5	5	6	*90*	*85*
Female region*	1	7	24	40	65	***99***
Species x region*	1	12	39	30	***96***	5
Male relative mass^a^	1	0	3	1	1	36
Trial number	1	2	1	.	0	.

Random subsampling from all trials created 1000 bootstrap replicates for interactions with conspecific males and interactions with heterospecific males that were statistically independent (see *Methods: Data analysis*). The values shown are the percentage of times that a fixed effect was significant in the resulting 1000 anova models (*italic P* ≤ 0.05 in ≥ 75%, ***bold italic****P* ≤ 0.05 in ≥ 95%). For time, visits, and mating attempts, female identity was included as a random effect (repeated measures analysis), which was not necessary for the female-averaged responses of affiliation and aggression. In full models, trial number and male relative mass (conspecific minus heterospecific male mass) were included as fixed effects, while reduced models omitted these 2 terms. For all 5 responses the reduced model set produced a better fit than the full model set (based on AICc), hence their percentages are shown (*).

^**a**^For affiliation and aggression, the average relative mass for the 2 sets of males tested with each female.

Aggression was more frequent in sympatric than allopatric females with both conspecific and heterospecific males (Figure 3E). However, the effect of region was stronger for interactions with heterospecific males than conspecific males (Table 1A,B, *P* ≤ 0.05 in 99% vs. 86% of models). Moreover, *N. lepida* females appeared to show more aggression with heterospecific males (Table 1B, *P* ≤ 0.05 for species in 85% of models) and *N. bryanti* females with conspecific males (*P* ≤ 0.05 for species in 75% of models). Both findings emphasize the role of large *N. bryanti* males. Likewise, the role of male relative mass was the strongest in the aggression models with heterospecifics, although significant (*P* ≤ 0.05) in only 36% of the bootstrap replicates.

### Net activity with conspecific males by female region and taxon

**Figure 4:**
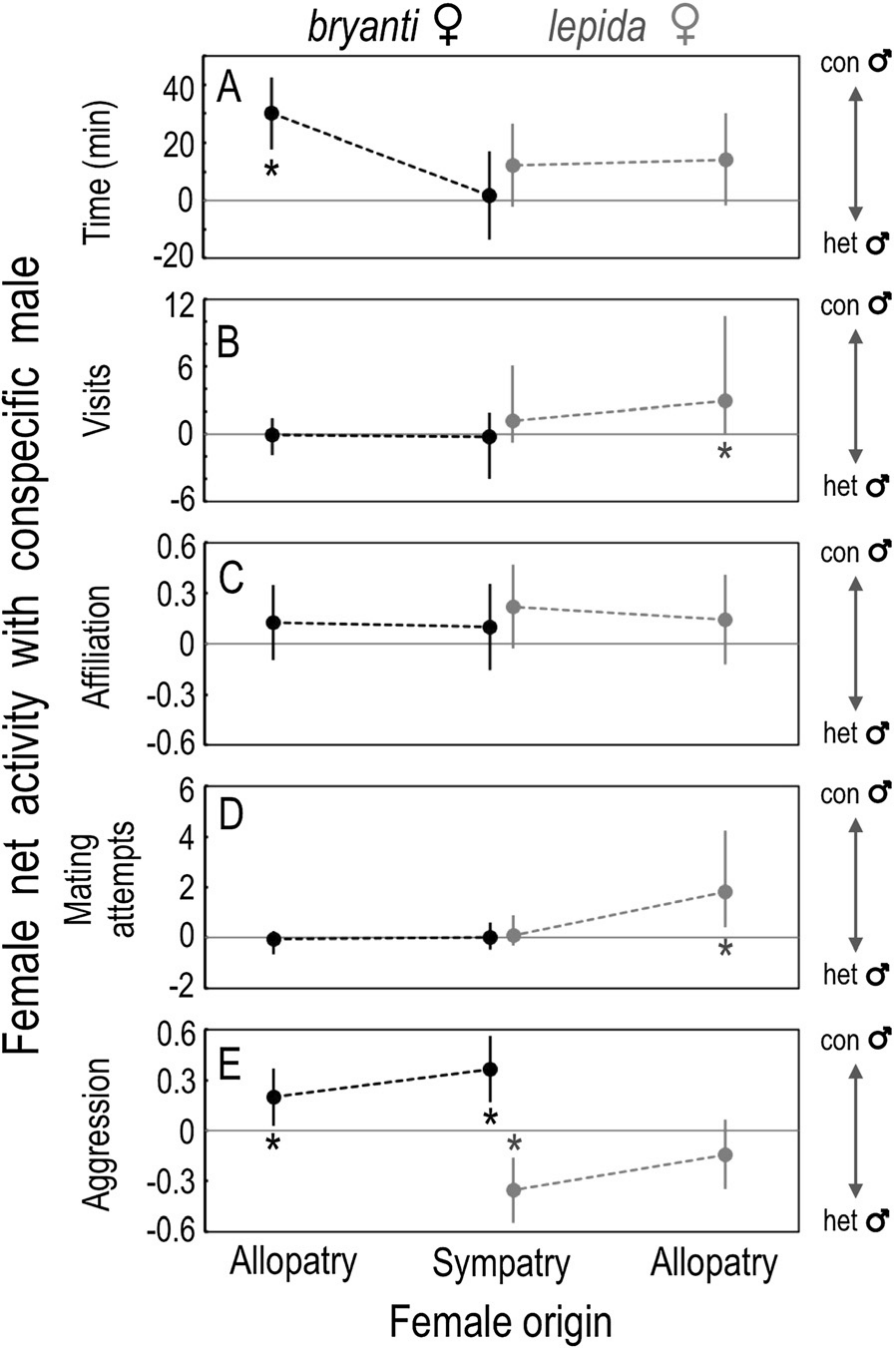
**The pattern of net activity with conspecific males in mate choice trials by female species and region.** The 5 responses include: **(A)** time with a male (entry tube plus cage, Figure 6), **(B)** visits to a male’s cage, **(C)** affiliation, **(D)** mating attempts, and **(E)** aggression. Responses **(C)** and **(E)** were the proportion of a female’s 2 trials where affiliation or aggression was observed. Each point represents the least squares mean difference (± 95% CI) between activity with conspecific and heterospecific males, with positive values indicating a preference for conspecifics. An asterisk (*) denotes a difference from zero (*t* test, *P* < 0.05). Statistical analyses on visits and mating attempts were performed on transformed values, but back-transformed values are shown (yielding asymmetric confidence intervals for these responses).

*Neotoma bryanti* and *N. lepida* females differed in their preference for conspecific males based on general and mating activity but not based on affiliative behavior. *Neotoma bryanti* spent more time with conspecific males in allopatry but not in sympatry, a regional change in preference not evident in *N. lepida* (Figure 4A; Table 2, *P* ≤ 0.077 for region and species-by-region). *Neotoma lepida* visited conspecific males more often than heterospecific males (Figure 4B; Table 2, *P* = 0.017 for species), a preference not evident in *N. bryanti*. *Neotoma bryanti* females showed no mating preference for conspecific males, while *N. lepida* showed a strong preference only in allopatry (Figure 4D; Table 2, *P* ≤ 0.041 for species and species-by-region). Both species showed more affiliative behavior with conspecific males, but the preference was not significant for either species in either region (Figure 4C).

**Table 2:** **Analysis of variance of the net activity of females with conspecific males for 5 responses: time, visits, affiliation, mating attempts, and aggression (*italic P *≤ 0.10, *bold P *≤ 0.05)**

		**Female net activity with conspecific males**									
		**Time**		**Visits**		**Affiliation**		**Mating attempts**		**Aggression**	
**Fixed effect**	**df**^a^	***F***	***P***	***F***	***P***	***F***	***P***	***F***	***P***	***F***	***P***
Female species*	1,61	0.1	0.709	6.1	**0.017**	0.3	0.587	7.3	**0.009**	30.3	**<0.001**
Female region*	1,61	4.3	**0.042**	0.5	0.489	0.0	0.840	1.2	0.270	0.1	0.818
Species x region*	1,61	3.2	*0.077*	0.1	0.838	0.2	0.688	4.4	**0.041**	3.8	*0.055*
Male rel. mass^b^	1,X^c^	0.1	0.785	1.1	0.300	0.9	0.352	0.9	0.345	1.2	0.275
Trial number	1,51^d^	0.6	0.450	0.0	0.859	.	.	0.1	0.744	.	.

Net activity was the response with the conspecific male minus that with the heterospecific male. Relative mass was the mass of the conspecific minus the heterospecific male. For time, visits, and mating attempts, female identity was included as a random effect (repeated measures analysis), which was not necessary for the female-averaged responses of affiliation and aggression. For all 5 responses, the reduced models (including only species, region, and their interaction) produced a better fit than the full models based on AICc, hence their *F* and *P* are shown (*).

^a^For df, both the numerator and denominator degrees of freedom are given for each effect.

^b^For affiliation and aggression, the average relative mass for the 2 sets of males tested with each female.

^c^The denominator degrees of freedom varied based on model structure and missing values. The values by response (left to right) were 51, 50, 60 (no repeated measures), 51, and 60 (no repeated measures), respectively.

^d^For visits, the denominator degrees of freedom were 50.

For emphasis, 0.05 ≤ *P* ≤ 0.10 are noted in *italics* and *P* ≤ 0.05 in **bold** type.

The analyses of net activity clearly show the difference in aggressive behavior by female species and male type (Figure 4E). *Neotoma lepida* were significantly more aggressive with heterospecific males and *N. bryanti* with conspecific males (Table 2, *P* < 0.001 for species). Moreover, this difference increased from allopatry to sympatry (Table 2, *P* ≤ 0.055 for species-by-region). In sum, interactions with *N. bryanti* males tended to be more aggressive in sympatry than in allopatry for both species of females.

### Body mass by species, sex, and region of origin

**Figure 5:**
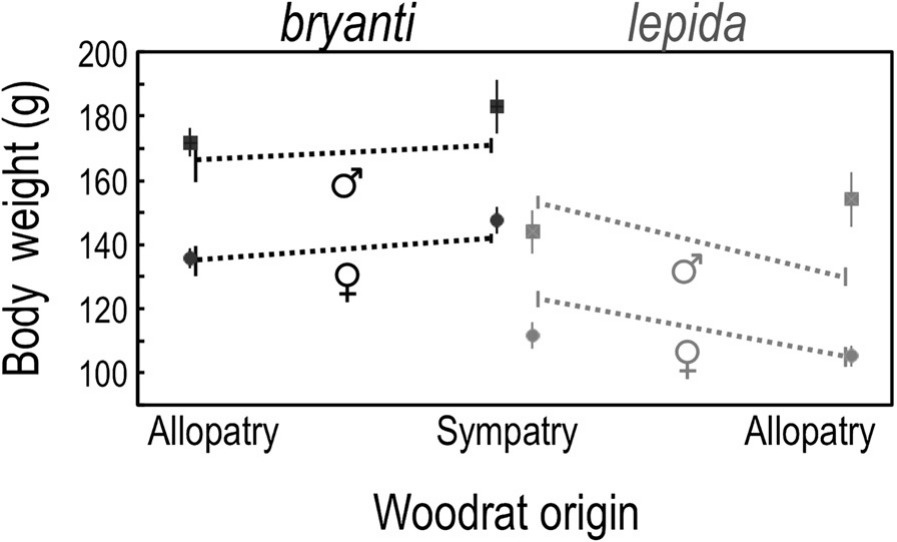
**Average mass (± 1 SE) of adult *N. bryanti *and *N. lepida *used in the mate choice trials (squares, males *n *= 33; circles, females *n *= 65) and from a broader sample (dashed lines linking thick bars).** The broader sample included trial animals plus additional adults from sympatry (*n* = 451; Whitney Well, Figure 1) and allopatry (*N. b.*, *n* = 6, from Alameda, Kern, and San Diego Counties; *N. l., n =* 86, from Inyo, Kern, Lassen, and San Bernadino Counties, Figure 1, inset). *Neotoma bryanti* were heavier than *N. lepida*, males were heavier than females, and mass increased in sympatry with a sharper increase in *N. lepida* (see *Results* for details).

The *N. bryanti* experimental animals were larger than *N. lepida* (*F*_1,90_ = 59, *P* < 0.001) and the males larger than females (*F*_1,90_ = 93, *P* < 0.001). These trends held for the broader sample that included field records from Whitney Well and the MVZ database (species: *F*_1,633_ = 100, *P* < 0.001, sex: *F*_1,633_ = 126, *P* < 0.001; Figure 5). We observed a marginal increase in mass from allopatry to sympatry (*F*_1,90_ = 2.8, *P* = 0.099) in the experimental animals, an increase that was significant under broader sampling (*F*_1,633_ = 26, *P* < 0.001) but more pronounced in *N. lepida* (region*species: *F*_1,633_ = 8.1, *P* = 0.005). No other terms were significant in either factorial analysis (*F*_1,90_ ≤ 2.4, *P* ≥ 0.121; *F*_1,633_ ≤ 0.6, *P* ≥ 0.455).

## Discussion

In the zone of secondary contact between *N. bryanti* and *N. lepida*, females have the potential to encounter heterospecific males that may be suitable mates based on certain traits, but with whom hybridization appears to be costly [23]. Using mate preference trials, we sought to assess whether, in addition to post-zygotic mechanisms (lower hybrid fitness), this system was also characterized by pre-zygotic behavioral mechanisms that would reduce gene flow between the species.

Our behavioral trials demonstrated that the preference for conspecific males was not higher in sympatry than in allopatry for either species of woodrat. These results contradict the classic expectations of reinforcement [12] in response to selection against hybridization. However, females of both species in sympatry were less likely to mate than were allopatric females following courtship, suggesting an increase in choosiness consistent with a higher selective cost of making a mating error in the contact zone. Moreover, sympatric females were more likely to have aggressive encounters with males than allopatric females, suggesting a heightened tendency to recognize males as competitors.

### Asymmetry in assortative mating

Our results show asymmetry between females of the two species in their degree of assortative mating. Because of the overall decline in mating activity in sympatry, the evidence for species-specific mate preferences is pronounced only in allopatric females: *N. lepida* preferred conspecific males, whereas *N. bryanti* showed no such preference (Figure 4D). *Neotoma bryanti* females, especially those from allopatric sites, often mated with both males in a trial, while *N. lepida* rarely exhibited such promiscuity (Figure 2B, dark bar sections). If *N. bryanti* prefer conspecifics but are adapted to breed with multiple males (e.g., [26]), they may have been more likely to mate with the heterospecific male after mating with the conspecific. However, no such ordinal pattern was evident; in trials with double matings, *N. bryanti* mated with the heterospecific male first in half of the trials.

Two species may come into secondary contact with behaviors and preferences that favor unidirectional introgression. For example, a preference for large or exaggerated traits (e.g., body size, signals) may predispose females of one species to choose heterospecific males if they possess more extreme traits than conspecifics [27]; reviewed in Pfennig 2007]. We have no evidence that female *N. bryanti*, *N. lepida*, or their congeners [28,29] prefer larger males or those with more extreme traits. However, the precopulatory behaviors of the two species were quite different in our trials, and may have been influenced by the body size difference of typical conspecific and heterospecific pairings. Before entering male cages, both allopatric and sympatric *N. lepida* females went back and forth between the entry tubes of both males more often than did *N. bryanti*, perhaps evaluating olfactory and visual cues. *Neotoma lepida* females also entered the cages of conspecific males significantly more than those of heterospecific males (Figures 3B, 4B), which tracked their mate preference. We suggest that the large size difference between *N. lepida* females and *N. bryanti* males (Figure 5) played a role in reducing heterospecific affiliation and increasing heterospecific aggression with these females (Figure 3C, 3E). Unlike *N. bryanti* females, *N. lepida* never overcame encounters that we scored as aggressive to reach copulation with *N. bryanti* males.

Preliminary genetic data suggest that hybridization is asymmetric in the field, and lies in the same direction as we observed experimentally; for all three F_1_ hybrid offspring with known parents (from Whitney Well, Figure 1), *N. bryanti* was the mother [30]. Beyond our hypothesis that behavior and size dimorphism across species plays a role in this asymmetry, we cannot rule out that genomic imprinting and/or other mechanisms may also favor directional hybridization [31]. Nor do we yet know how the survivorship and fecundity of hybrids with *N. bryanti* mothers compares to offspring from reciprocal heterospecific pairings. Pfennig [32] found that asymmetric hybridization is favored in spadefoot toads under conditions (shallow ponds) where the traits of hybrid offspring are favored (faster larval development). Because the woodrat contact zone occurs at a sharp ecotone between woodland and desert scrub, offspring of *N. bryanti* females may benefit from traits carried by *N. lepida* which are better adapted to desert conditions, particularly in dry years. Although similar arguments could be made favoring introgression in the opposite direction (*i.e*., for *N. lepida* to “capture” *N. bryanti* traits, that may impart larger body size and greater competitive ability in their offspring), *N. lepida* females may typically be intimidated from reaching advanced courtship and copulation with *N. bryanti* males due to their large size and observed tendency to be more aggressive.

### Decrease in mating activity in sympatry

Despite the lack of evidence for an increase in assortative mating, females of both species from sympatric sites were less likely to mate during the two-hour trial period than were allopatric females (Figure 2B). At least three factors may explain this pattern. First, the decrease in mating activity in sympatry may result from selection favoring increased aggression, which ultimately interferes with courtship. Aggression increased in sympatry between females and both species of males (Figures 2C, 3E), matching the decrease in observed mating activity (Figure 3D). In woodrats, “boxing”, a forward-facing, paw-to-paw jabbing by participants, often precedes lordosis by females and copulation [33] but can also lead to aggressive encounters [34] such as chasing or biting by either the female or the male, and retreat by one or both parties. Importantly, aggressive encounters were more common with sympatric *N. bryanti* males, meaning that *N. bryanti* females were subject to more aggression with conspecific males in sympatry, and *N. lepida* females with heterospecifics (Figure 3E). Nishikawa [35] and Deitloff et al. [36] observed a similar increase in interspecific aggression in sympatry in two different species pairs of *Plethodon* salamanders. The pattern we observed in our trials is consistent with one wherein both species of woodrats in sympatry selectively benefit from increased aggression, perhaps because it increases their ability to compete for optimal den sites, especially the relatively rare boulder den sites [20]. As suggested by Peiman and Robinson [37,38], the pattern should be more pronounced with the dominant species, as we observed in interactions with larger *N. bryanti* males (Figure 4E).

We suggest that territorial interactions in sympatry may also selectively favor larger body size, particularly in the subordinate species. Based on a combination of our field data and that of others from the Museum of Vertebrate Zoology database, we found a significant increase in adult body size in *N. lepida* and a marginal increase in *N. bryanti* in the zone of sympatry (Figure 5). A similar increase and convergence in adult body mass was observed between *Neotoma macrotis* and *Neotoma fuscipes* in the Sierra Nevada [39], also two species exhibiting strong territoriality around denning sites. In areas of multi-species overlap in woodrats, large-bodied species outcompete smaller-bodied species for access to optimal den sites [20]. It is reasonable to expect that when high quality den sites, such as those in boulder outcrops, are sparsely distributed across the landscape, there will be strong competition to occupy these sites, potentially selecting for increased body size and competitive behaviors (aggression). Body size in woodrats is certainly also responsive to other factors, most notably environmental temperature [40], and changes in this trait would alter competitive abilities in areas of sympatry.

A second explanation for the dip in mating activity in sympatry, and not exclusive to the first explanation of augmented aggressive tendencies, may be increased choosiness by sympatric females. Each trial offered females only two choices, with sympatric females more likely to reject both. Consistent with an increase in choosiness, when courtship occurred (tail rattles, mutual grooming, boxing, and/or lordosis) without observed aggression, sympatric females were still less likely to mate than were allopatric females. This pattern was stronger in *N. bryanti* but numerically there were more trials in which *N. lepida* courted but did not mate. In allopatric populations where we measured relatively high rates of mating, such behavior began within the first hour in over 95% of the trials. While our trial length appeared adequate for mate choice in these females, it may have been inadequate for pairs from sympatric locations. Successful pairing in sympatric populations may require more time for mate inspection and evaluation than our trials allowed. If true, this would suggest that a female’s prior knowledge of her potential mates may be particularly important in contact zones. Even in non-contact zone woodrat populations, females typically mate with 1 or more neighboring males [41,42], individuals that she has been living near and presumably had an opportunity to inspect. Especially in an environment where aggression and territoriality may be heightened (this study), established neighbor relationships may be particularly important in mediating aggression and making mating decisions.

A third hypothesis to explain the lower rates of copulation by sympatric females is that some of these females may have been in poor condition. Of the sympatric females trapped in our first trip, 15% of the *N. bryanti* and 0% of the *N. lepida* copulated in their first trial in captivity, but the rate of mating by these females increased to 38-40% in their second trial ≥ 90 days after being fed *ad libitum* in the lab. The lower likelihood of mating for females trapped during the first sampling trip compared to the 2nd and 3rd trips was significant. However, even after excluding these females, sympatric females mated less often than allopatric females, suggesting that the condition of these females did not explain the greater reluctance of sympatric females to mate. While our results are robust to the immediate post-field condition of these females, their initial reluctance to mate raises an important issue concerning the overall condition of sympatric versus allopatric animals. In this system (as in many) the sympatric populations exist at the edge of their respective species’ ranges where environmental conditions may be suboptimal, even in the absence of a heterospecific. While captive conditions would quickly equalize some aspects of condition in test subjects, there may be lingering effects of poor field conditions that are a true reflection of life in range-edge habitats. If, on average, individuals from range-edge populations exist in relatively poor condition that contributes to low motivation to mate, this may be an important factor determining population dynamics and the potential for interspecific gene flow.

## Conclusions

Our previous work in this system provided evidence of pre-zygotic isolation due to fine-scale habitat segregation that limits interspecific mating opportunities, and post-zygotic isolation due to apparent low hybrid survival in the early stages of life [23]. In contrast, the current analysis reveals behavioral interactions that would seem to increase the potential for, at least, unidirectional gene flow between the species. Specifically, our analyses show that *N. lepida* females from allopatric populations show a preference for conspecifics, whereas allopatric *N. bryanti* females are equivocal in their choice of mates. Although these preferences may be due to species-specific mate recognition, the larger body size and greater aggressiveness of *N. bryanti* males likely play central roles in *N. lepida* mate preference. That is, *N. lepida* females may have a negative response to the much larger *N. bryanti* males, whereas *N. bryanti* females may not be similarly deterred by the smaller, relatively docile *N. lepida* males. Because female *N. lepida* have a clear preference for conspecifics in allopatry, it is likely that such females would have come into secondary contact with this predisposition, and that this preference would have limited direct interspecific matings and gene flow. In contrast, given patterns in allopatry, *N. bryanti* females likely came into secondary contact amenable to mating with heterospecifics, a behavior promoting interspecific gene flow.

The intensity of aggression exhibited among sympatric animals was so pronounced as to make assessment of mate preferences difficult. However, even in trials where aggression did not occur, sympatric females still mated less often, which may indicate increased choosiness or decreased sexual motivation in these animals. While field observations of aggression and mating patterns are needed to confirm the findings of our laboratory trials, body size and aggression likely play key roles in the rate of interspecific gene flow in this system.

While we have yet to model evolutionary outcomes that consider the behavioral barriers (or lack thereof) that we have described, all else being equal, if post-zygotic isolation is strong, *N. bryanti* would appear to be at a potential fitness disadvantage. However, there appear to be relatively few opportunities for the species to interbreed because of their strong ecological segregation. As such, the potential evolutionary impact of the behavioral patterns observed in the laboratory may not be fully realized in a natural field setting because of the over-riding role of ecological segregation. Adequately modeling the dynamics of this zone will require a more complete understanding of the mate choice patterns and reproductive success of hybrids that survive to adulthood.

In conclusion, this system provides a new example of multiple pre- and post-zygotic isolating mechanisms operating simultaneously. The *N. bryanti*/*N. lepida* hybrid zone offers the potential to further understand how natural and sexual selection maintain reproductive isolating mechanisms, and how these mechanisms interact to inhibit or, in some cases, facilitate interspecific gene flow.

## Methods

### Field collections and captive husbandry

We collected woodrats from allopatric and sympatric localities in the Sierra Nevada foothills of northeastern Kern County, California (Figure 1) during three separate sampling trips. We collected at allopatric sites characterized by Patton et al. [43] as maintaining either pure *N. lepida* (Freeman Canyon) or *N. bryanti* (Walker Canyon). Approximately equidistant (~25 km) between these allopatric localities, we collected pure *N. lepida* and *N. bryanti* from five populations known to harbor both species and/or hybrids (Figure 1; Well Rd, Hoffman Summit, Piute Mt. Road, and near Whitney Well; the latter being the site of a recent 4-year demographic study [23]. Species identity was confirmed with 15-locus microsatellite genotypes [23]. All activities were conducted under a scientific collecting permit from the California Department of Fish and Game, protocols approved by the Idaho State University Institutional Animal Care and Use Committee, and in accordance with standards outlined by the American Society of Mammalogists [44].

Woodrats were individually housed, initially in standard rat cages (43 × 24 × 20 cm) with air filter lids while in quarantine, and then transferred to rectangular wire mesh cages (34 by 28 cm), either 37 or 51 cm high, with plastic bottoms that allowed vertical climbing onto small shelves within the cages. Male and female cages were spatially segregated; animals were fed Harlin Tech rabbit and rodent pellets *ad libitum*, and given small slices of carrot daily. As a retreat, each animal was provided with a 15 cm long (10 cm diameter), plastic tube, capped at one end. Room temperature was set at 23°C and relative humidity at 30%. The room was lit with GE Chroma 50 bulbs, on a light–dark schedule corresponding to the collection site on June 1 to simulate conditions at the height of the mating season.

### Experimental procedure

Because no external signs of estrus are evident in woodrats, to bring females into a behavioral estrus, we injected two hormones prior to experimentation, as done in previous studies [45-47]. Forty-six to 50 hours prior to a trial, we injected females subcutaneously with a compound of estradiol benzoate and peanut oil (1 mg/ml) at a dose of 0.1 ml/100 g of body weight. Four to six hours pre-trial, we injected females with a compound of progesterone and peanut oil (10 mg/ml) at a dose of 1 ml/100 g of body weight. In tests of this procedure prior to our experiments, 4 of 5 treated females copulated with males when housed together for a 2 hour period.

**Figure 6:**
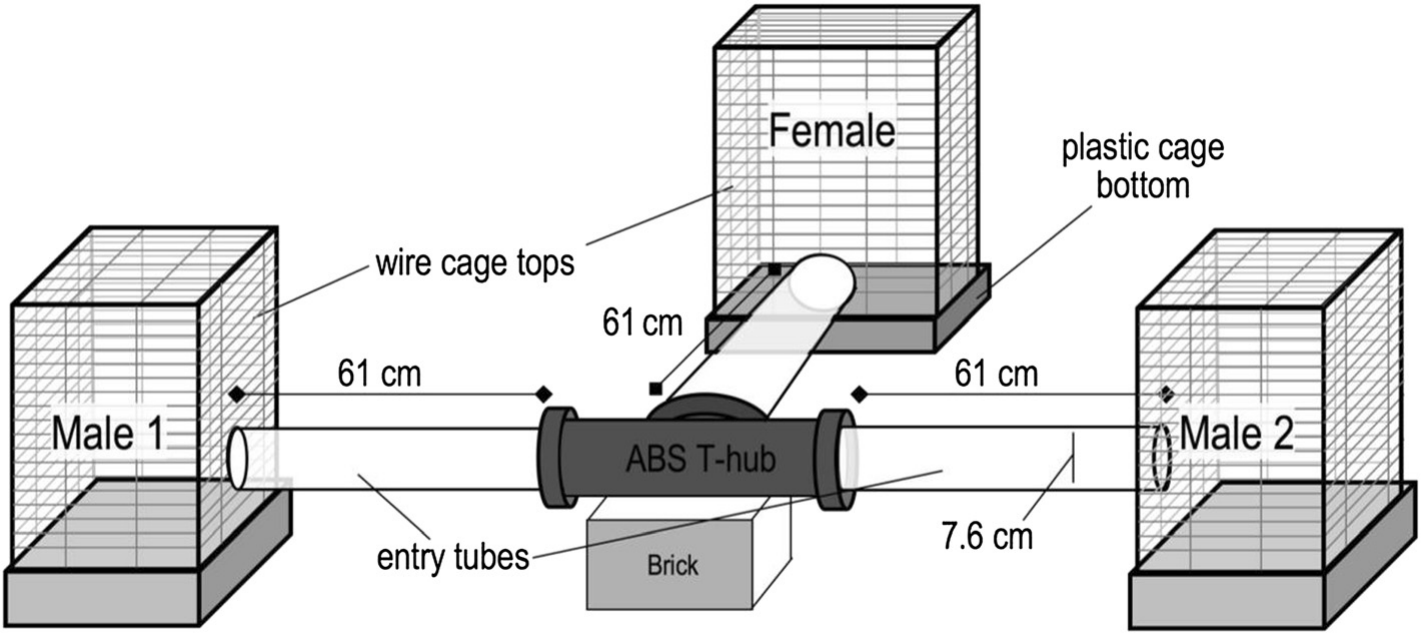
**T-maze used for paired choice experiments, consisting of 3 clear acrylic tubes joined by an ABS T-hub.** During a trial, females could move freely between cages, while each male was limited to his cage via a tether (see *Experimental procedure*). Female time spent with males (Figures 3, 4) included time in male cages and in 'entry tubes’, where females would often sit with their heads near the cage entrance.

Once in behavioral estrus, we offered females a choice of two males– one conspecific, one heterospecific – using a T-maze (Figure 6) modified from Smadja and Ganem [48]. Females were randomly selected from either allopatric or sympatric populations. Males of each species were selected randomly from those captured > 225 m from the test female, to minimize the possibility of testing neighbors or individuals previously familiar with one another. We weighed males and females pre-trial, but males within a trial were not matched by mass, as *N. bryanti* are nearly always heavier than *N. lepida* (see *Results: Body mass*).

Each female was tested in two trials, with the second trial run ≥ 14 days after the first. Although males were used in several trials, a female had different males for each trial. Male cage position was counterbalanced such that conspecific males were on the left for half of the trials. Bedding in cages of participants was not changed within 48 hours prior to a trial. Within the T-maze (Figure 6), a female could move freely to either male’s cage and could assess male olfactory cues within the tubes prior to entering cages. Each male was restricted to his cage by a tether attached to a modified ferret harness, although he had full access to his cage floor. It was necessary to restrain males because our objective was to quantify female behavior without the confounding influence of male-male interactions. Previous experiments in which two heterospecific males were confined to an arena nearly always resulted in violent encounters and dominance by *N. bryanti* (Q. Shurtliff, unpublished data).

We ran each trial for two hours within the first 5-6 hours of the daily dark cycle. Two camcorders equipped with infrared technology were set on tripods at a height of 1 m, each placed ~1 m from a male’s cage. The viewing frame included the male’s cage, the tube leading from his cage to the ABS T-hub, and the female’s exit tube and cage (Figure 6). After tethering the males, the room was darkened except for a single red light and the test female’s cage was then connected to the maze. The trial began when the female entered the exit tube from her cage (usually ≤ 10 minutes after setup) or began by default if she had not left her cage after 20 minutes. At the end of each trial, the T-maze was cleaned with mild detergent, rinsed, and dried.

While conducted in a laboratory setting, we aimed to capture fundamental features of intersexual interactions between woodrats in the wild including 1) adults are territorial and maintain a primary midden or house (simulated by single occupancy of cages), which is also likely the site of mating (Matocq, pers. obs.), 2) females have an opportunity to visit and compare neighboring males, as in our choice experiment, and 3) females choose their mates, which can include the choice of mating with multiple males as evidenced by multiple paternity in the wild [29,41].

### Data analysis

#### Behavioral interactions: the effects of female taxon in allopatry and sympatry

For each behavioral trial, we recorded: 1) the time spent in the tube leading to a male’s cage and in his cage, often the only measure of preference included in mate preference studies [48]; 2) the number of visits to a male’s cage; 3) the presence or absence (1 or 0) of social-affiliative behavior between a male and a female, instigated by either sex; 4) the number of mating/copulation attempts; and 5) the presence or absence (1 or 0) of aggression between a male and a female, instigated by either sex. Affiliative behavior included tail rattles, mutual grooming and sniffing, female submission (or lordosis), and boxing. Boxing, in which the sexes face each other and jab with their paws, was classified as affiliative not agonistic (unlike in [34]), because it frequently precedes copulation in woodrats [33]. Aggression included scratching, biting, lunging, chasing, and the balling-up of either the female or the male [33,34].

Initially, we used contingency tests (*G*-tests) to assess if the occurrence of affiliative behavior, mating/copulation, or aggression (irrespective of the chosen male) depended on a female’s region of origin (allopatry or sympatry) or species (*N. bryanti* or *N. lepida*). We assessed the effect of female taxon and region using a Cochran-Mantel-Haenszel statistic (C-M-H, denoted $\chi_{\mathrm{cmh}}^{2}$, df = 1) and female trial number using Breslow-Day tests ($\chi_{\mathrm{BD}}^{2}$, df = 1). Some females exhibited mating activity with both males; therefore, we repeated the first set of analyses to assess how the likelihood of such dual activity depended on region and species. Finally, because we trapped females during different collecting trips and conducted trials year round, we used logistic regression to test whether the trapping period affected the likelihood of mating.

Beyond the simple occurrence of behavior, females may differ by species and region in the intensity of their activity with conspecific and heterospecific males. We used linear models to assess such variation in 5 responses with each class of male: 1) time with a male in his entry tube plus cage (Figure 6); 2) number of visits to a male’s cage; 3) average occurrence of affiliative behavior (*i.e.,* for each class of male, the proportion of a female’s two trials where affiliation was observed, either 0, 0.5, or 1.0); 4) number of mating/copulation attempts; and 5) average occurrence of aggression (calculated as for affiliative behavior). In any given trial, the interaction a female has with one male is not independent of her interaction with the other. Hence, to assess female activity with each class of male using independent observations, we subsampled from our set of 118 trials (e.g., [49]). Each sample randomly picked the conspecific as the focal male in half of the trials and the heterospecific in the other half. We repeated this process to produce 1000 subsamples (bootstrap replicates) for each class of male.

We analyzed each bootstrap replicate using repeated measures anova (mixed procedure, SAS v. 9.2) by species, region, and their interaction, including female identity as a random effect (each female was present in 1-2 trials per replicate). The models for affiliation and aggression were not repeated measures because these responses were averages for each female across her trials. Because relative body size might be key to female preference [50], we included a male’s relative mass (conspecific minus heterospecific mass) as a covariate. Model effects were considered significant if their test statistic was significant (*P* ≤ 0.05) in ≥ 95% of the models [51]. We also ran reduced models for each response, omitting trial number and male relative mass as fixed effects (but including female identity as a random effect where appropriate), and compared their fit with the full models. We report results for a reduced model set when its mean Akaike Information Criterion (AIC; i.e., the mean of 1000 AICc values) was lower than that for the corresponding set of full models. For all analyses, counts were square-root transformed and proportions arcsine square-root transformed prior to analysis.

Finally, to assess whether females interact more with conspecific males in sympatry than in allopatry, we calculated net conspecific responses for each trial that were the difference in each response (time, visits, affiliation, mating attempts, and aggression) between the conspecific and heterospecific male. We used repeated measures anova to see how these net responses varied by female species, region, and their interaction. Female identity was included as a random effect except for in models of net affiliation and aggression (as described previously, only one value per female). As above, we included male comparative weight (conspecific minus heterospecific) as a covariate. We again ran reduced models, omitting male relative weight and trial number as fixed effects, and report their results if they fit better (lower AICc) than the corresponding full model. For the net responses for time, affiliation, and aggression, the raw difference between conspecific and heterospecific male values produced normal residuals. For net visits and mating attempts, the difference between square-root transformed counts produced normal residuals.

### Regional patterns in body size

To identify any changes in body size between allopatry and sympatry, we examined individual body size (*i.e.*, weight) by region, species, and sex using factorial ANOVA. We assessed trends in body size at two spatial scales: 1) across the region of collections for this study (Figure 1), and 2) across a broader range within both species. For the broader range analysis, we included animals from our experimental behavioral trials, sympatric animals from our demographic study site at Whitney Well (Figure 1), and specimens with mass data from the Museum of Vertebrate Zoology (Berkeley, CA) from Kern and five other California counties (Figure 1, inset).

## Competing interests

The authors declare that they have no competing interests.

## Authors’ contributions

QRS helped design the study, obtained partial funding, conducted all field collections and behavioral trials, participated in data analysis, and contributed to writing the manuscript. PJM conducted data analysis and contributed to writing the manuscript. JDY assisted in behavioral trails. MDM helped design the study, obtained partial funding, assisted in data analysis and contributed to writing the manuscript. All authors read and approved the final manuscript.
